# Psychiatric manifestations of anti-NMDAR encephalitis

**DOI:** 10.1192/j.eurpsy.2021.405

**Published:** 2021-08-13

**Authors:** D. Martins, R. Faria, S. Rodrigues, M. Pinho

**Affiliations:** 1 Department Of Psychiatry, Hospital de Magalhães Lemos, Porto, Portugal; 2 Department Of Child And Adolescent Psychiatry, Centro Hospital e Universitário do Porto, Porto, Portugal

**Keywords:** anti-NMDAR encephalitis, psychiatry, psychiatric manifestations

## Abstract

**Introduction:**

Anti-N-methyl-D-aspartate receptor (NMDAR) encephalitis is an autoimmune disorder characterized by neuropsychiatric symptoms before progressing to seizures, complex movement disorder, autonomic dysfunction and hypoventilation.

**Objectives:**

Presenting a review of the psychiatric manifestations of anti-NMDAR encephalitis.

**Methods:**

Search on Pubmed® and Medscape® databases with the following keywords: “psychiatric”, “anti-NMDA receptor encephalitis” and “anti-NMDAR encephalitis”. We focused on data from systematic reviews and meta-analyzes. The articles were selected by the authors according to their relevance.

**Results:**

Studies show that 77% to 95% of patients with anti-NMDAR encephalitis initially present psychiatric manifestations. Age and sex distribution are young women, and the frequency of cases is lower after 40 years of age. The most common psychiatric symptoms are agitation (59%) and psychotic symptoms (54%). The psychotic symptoms more common are visual (64%), auditory (59%) hallucinations and persecutory delusions (73%). Catatonia is described in 42% of patients. Antipsychotic treatment induces an adverse drug reaction (33%), the neuroleptic malignant syndrome represents 22% of the cases. Delays in distinguishing this disease from a psychiatric disorder can have serious complications, with a mortality of up to 25% in patients receiving limited or delayed immunotherapy.

**Conclusions:**

It’s important to consider anti-NMDAR encephalitis in the differential diagnosis of patients with an acute onset psychosis or unusual psychiatric symptoms. Antipsychotic treatment should be use with caution when suspected or confirmed anti-NMDAR encephalitis. Without appropriate treatment, patients may suffer a protracted course with significant long-term disability or death. A clinical index of suspicion is required to identify patients who would benefit from cerebrospinal fluid testing and immunotherapies.

**Disclosure:**

No significant relationships.

